# Multiorbital charge-density wave excitations and concomitant phonon anomalies in Bi_2_Sr_2_LaCuO_6+δ_

**DOI:** 10.1073/pnas.2001755117

**Published:** 2020-06-25

**Authors:** Jiemin Li, Abhishek Nag, Jonathan Pelliciari, Hannah Robarts, Andrew Walters, Mirian Garcia-Fernandez, Hiroshi Eisaki, Dongjoon Song, Hong Ding, Steven Johnston, Riccardo Comin, Ke-Jin Zhou

**Affiliations:** ^a^Diamond Light Source, Harwell Campus, Didcot OX11 0DE, United Kingdom;; ^b^Beijing National Laboratory for Condensed Matter Physics and Institute of Physics, Chinese Academy of Sciences, Beijing 100190, China;; ^c^Department of Physics, Massachusetts Institute of Technology, Cambridge, MA 02139;; ^d^National Synchrotron Light Source II, Brookhaven National Laboratory, Upton, NY 11973;; ^e^H. H. Wills Physics Laboratory, University of Bristol, Bristol BS8 1TL, United Kingdom;; ^f^National Institute of Advanced Industrial Science and Technology (AIST), Tsukuba, Ibaraki 305-8560, Japan;; ^g^Department of Physics and Astronomy, The University of Tennessee, Knoxville, TN 37996

**Keywords:** charge-density waves, resonant inelastic X-ray scattering, phonon anomaly, high-temperature superconducting cuprates

## Abstract

Charge-density waves (CDWs) are ubiquitous in underdoped cuprate superconductors. As a modulation of the valence electron density, CDWs in hole-doped cuprates possess both Cu-3*d* and O-2*p* orbital character owing to the strong hybridization of these orbitals near the Fermi level. Here, we investigate underdoped Bi_2_Sr_1.4_La_0.6_CuO_6+δ_ using resonant inelastic X-ray scattering (RIXS) and find that a short-range CDW exists at both Cu and O sublattices in the copper-oxide (CuO_2_) planes with a comparable periodicity and correlation length. Furthermore, we uncover bond-stretching and bond-buckling phonon anomalies concomitant to the CDWs. Comparing to slightly overdoped Bi_2_Sr_1.8_La_0.2_CuO_6+δ_, where neither CDWs nor phonon anomalies appear, we highlight that a sharp intensity anomaly is induced in the proximity of the CDW wavevector (Q_CDW_) for the bond-buckling phonon, in concert with the diffused intensity enhancement of the bond-stretching phonon at wavevectors much greater than Q_CDW_. Our results provide a comprehensive picture of the quasistatic CDWs, their dispersive excitations, and associated electron-phonon anomalies, which are key for understanding the competing electronic instabilities in cuprates.

## Significance

Charge-density waves (CDWs) are a ubiquitous form of electron density modulation in cuprate superconductors. Unveiling the nature of quasistatic CDWs and their dynamical excitations is crucial for understanding their origin––similar to the study of antiferromagnetism in cuprates. However, dynamical CDW excitations remain largely unexplored due to the limited availability of suitable experimental probes. Here, using resonant inelastic X-ray scattering, we observe dynamical CDW excitations in Bi_2_Sr_2_LaCuO_6+δ_ (Bi2201) superconductors through its interference with the lattice. The distinct anomalies of the bond-buckling and the bond-stretching phonons allow us to draw a clear picture of funnel-shaped dynamical CDW excitations in Bi2201. Our results of the interplay between CDWs and the phonon anomalies shed light on the nature of CDWs in cuprates.

Cuprate superconductivity is achieved through doping into the stoichiometric parent compound, which is a charge-transfer Mott insulator with strong electronic correlations. Here, the injected holes can segregate into periodically spaced domain walls, rapidly suppressing static antiferromagnetic (AFM) order. A classic example is the ordered stripe phase in La_1.48_Nd_0.4_Sr_0.12_CuO_4_ (LNSCO), where both spin and charge form a quasistatic order near a doping level of ∼1/8 (1). Recently, quasi–two-dimensional (2D) short-range charge-density waves (CDWs) have been discovered in most hole-doped cuprates (2–12). Although hole carriers are primarily doped into the O-2*p* orbitals in the CuO_2_ planes, resonant elastic X-ray scattering (REXS) experiments have detected the quasistatic CDW order within both the Cu and O sublattices in La_1.875_Ba_0.125_CuO_4_ (LBCO) owing to the strong hybridization between the Cu-3*d* and the O-2*p* orbitals (2, 13, 14). The CDW in LBCO is, however, relatively long-range, leaving an open question on whether generic short-range CDWs (such as the Bi-based compounds) also project onto multiple orbitals.

Subsequent to the discovery of the stripe phase in LNSCO, inelastic neutron scattering (INS) identified a pronounced softening in the Cu-O bond-stretching phonon branch close to the CDW wavevector in La-based cuprates and YBa_2_Cu_3_O_6+δ_ (YBCO) (15). In Bi_2_Sr_2_LaCuO_6+δ_, nonresonant inelastic X-ray scattering (IXS) studies also revealed softening in the bond-stretching phonon (16). Most recently, a similar electron-phonon anomaly was uncovered by resonant inelastic X-ray scattering (RIXS) in underdoped Bi_2_Sr_2_CaCu_2_O_8+δ_ (Bi2212), where a short-range CDW occurs (17). These studies suggest an intimate link between electron-phonon coupling (EPC) and CDW correlations. Interestingly, a dynamical dispersive CDW excitation is inferred in Bi2212 as an anomalously enhanced phonon intensity upon the interference between phonons and underlying charge excitations (17). It is unclear, however, whether such dispersive CDW excitations are ubiquitous to other cuprate families.

To date, the underlying mechanism of CDWs in cuprates still remains elusive. Unveiling the nature of the quasistatic CDWs as well as their dynamical excitations is crucial for understanding the CDW’s origin––similar to the study of AFM in cuprates. Although the quasistatic properties of CDWs have been investigated extensively, their dynamics (i.e., the collective CDW excitations) are largely unexplored due to the limited availability of suitable experimental tools. RIXS is one of the few techniques that directly probe quasistatic CDWs, their excitations, and electron-phonon coupling, which are important elements for identifying the CDW mechanism.

## Results and Discussion

**Fig. 1.**
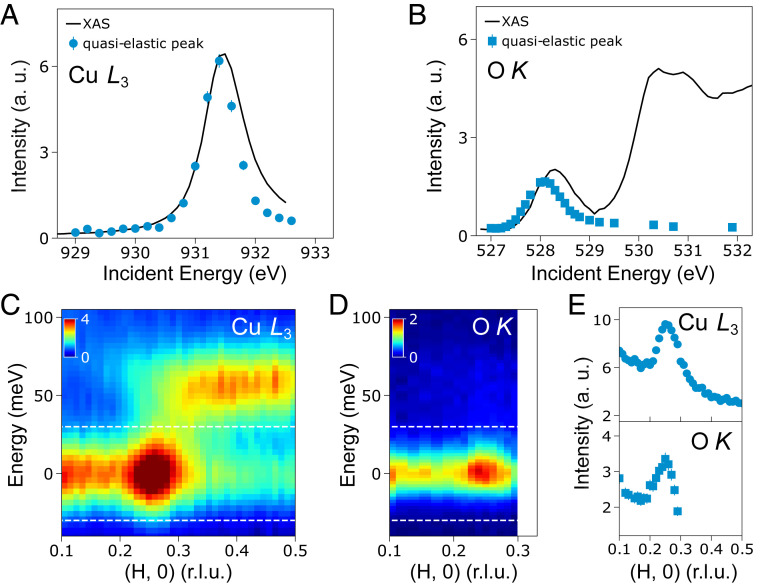
CDW in underdoped UD23. (*A* and *B*) Solid black lines: Cu *L*_3_- and O *K*- edges XAS of UD23 sample collected with σ incoming polarization in normal incidence geometry (see the experimental setup in *SI Appendix*, Fig. S2). Blue circles (squares): Energy-dependent scattering intensities of the quasielastic peaks at the Cu *L*_3_-edge (O *K* edge) at *q*_//_ = 0.26 r.l.u. (0.25 r.l.u.). (*C* and *D*) RIXS intensity maps excited at, the Cu *L*_3_ resonance of 931.6 eV and the O *K*-edge hole-peak resonance of 528.4 eV as a function of energy and *q*_//_ along the (H, 0) direction. (*E*) Integrated intensity of the quasielastic peak, at the Cu *L*_3_- and O *K* edges, within an energy window (±30 meV) marked by the two white dashed lines in *C* and *D*.

We initially used RIXS to study an underdoped superconducting single-layer Bi_2_Sr_1.4_La_0.6_CuO_6+δ_ (Bi2201) (superconducting temperature T_*c*_ = 23 K, UD23) (*Materials and Methods* and *SI Appendix*, Fig. S1). Fig. 1 *A* and *B* show the Cu *L*_3_ and O *K* X-ray absorption spectra (XAS) of UD23 with the incoming linear polarization (σ) parallel to the CuO_2_ planes (σ polarized light is used throughout unless otherwise stated) (see *SI Appendix*, Fig. S2 for the experimental geometry). The Cu *L*_3_ and O *K* XAS represents a projection of the unoccupied states of planar Cu-3*d* and O-2*p* orbitals, respectively. The momentum dependence of the Cu *L*_3_ RIXS measurements was acquired at the Cu *L*_3_ resonance (*E* ∼ 931.6 eV) and over a broad range of in-plane momentum transfers *q*_//_. Fig. 1*C* presents the low-energy excitations (<100 meV) in the Cu *L*_3_ RIXS spectra as a function of *q*_//_ = (H, 0). A quasielastic scattering peak is clearly visible at *q*_//_ ∼ 0.25 reciprocal lattice units (r.l.u.). Such a peak was also seen in RIXS spectra under different configurations (*SI Appendix*, Fig. S3). O *K* RIXS spectra were collected with incident photon energy tuned to the resonance of the mobile carrier (hole) peak (*E* ∼ 528.4 eV) as a function of *q*_//_ = (H, 0) and up to ∼0.3 r.l.u. Fig. 1*D* shows the corresponding data, where a quasielastic scattering peak also appears at *q*_//_ ∼ 0.25 r.l.u.

To clarify the origin of these peaks, we performed an energy-dependent RIXS scan at a fixed q_//_ at both resonances (Cu *L*_3_ and O *K*). The integrated intensities of the quasielastic peak are superimposed on top of the XAS data in Fig. 1 *A* and *B*. The resonance enhancement of the intensities at the Cu *L*_3_- and O *K* hole peaks unambiguously proves the existence of a CDW on both the Cu and O sublattices of the CuO_2_ planes (2, 6, 11, 14). Notably, an energy offset exists between the maximum of the XAS spectra and the peak of the energy-dependent scattering intensities of the quasistatic CDW. A comparable offset has been seen in multiple cuprate CDW studies where the XAS connects with the imaginary part of the atomic form factor while the scattering relates to the real part of the form factor (2, 14, 18). For a more quantitative assessment, we plot the integrated intensity of the quasielastic peak in Fig. 1*E*. The peak position of the CDW is extracted to be ∼0.259 ± 0.006 r.l.u. and ∼0.25 ± 0.003 r.l.u. at Cu *L*_3_- and O *K* edges, respectively. The full width at half-maximum (FWHM) of the CDW peak is Γ ∼ 0.078 ± 0.006 r.l.u. at the Cu *L*_3_ and Γ ∼ 0.076 ± 0.006 r.l.u. at O *K* edges. The corresponding CDW correlation length is 2/ Γ ∼ 15 Å. Note that a comparable difference of the CDW wavevector between the Cu and O sublattices was also observed in REXS results from LBCO and is due to a tiny change in the refractive index of X-rays between the O *K-* and the Cu *L*_3_ edges (2). We confirmed these observations on a second sample of UD23 and obtained comparable results (*SI Appendix*, Fig. S4). The in-plane CDW wavevector and the FWHM of the CDW peak at the Cu *L*_3_ are consistent with data obtained on similar Bi2201 compounds (6, 11). We conclude that the observed CDWs at the Cu and O sublattices have comparable periodicity and correlation length, reflecting the projection of the same electronic order onto multiple orbitals.

**Fig. 2.**
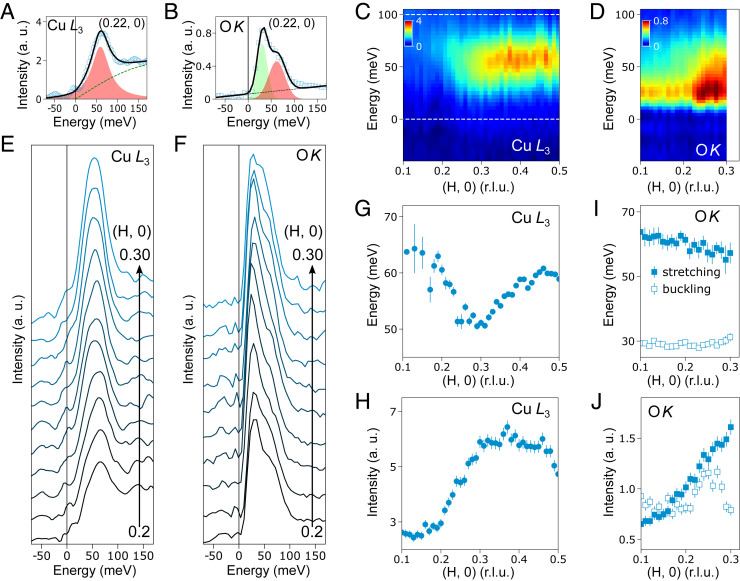
Phonons in underdoped UD23. (*A* and *B*) Fitting examples of the inelastic excitation at the Cu *L*_3_- and O *K* edges. Fitting details are described in *Materials and Methods*. (*C* and *D*) Cu *L*_3_- and O *K* RIXS intensity maps as shown in Fig. 1 *C* and *D* with the fitted elastic peak subtracted. (*E* and *F*) Cu *L*_3_- and O *K* RIXS spectra after removing the fitted elastic peaks at selected momentum transfer values ranging from *q*_//_ = 0.2 to 0.3 r.l.u. along the (H, 0) direction. (*G* and *H*) The fitted dispersion and the integrated intensity of the bond-stretching phonon excitations at the Cu *L*_3_ edge as a function of momentum. The phonon spectral weight is integrated within the energy window (0–100 meV) illustrated by the white dotted line in *C*. (*I* and *J*) The dispersion and integrated intensity of the bond-stretching phonon (the high-energy peak, filled markers) and the bond-buckling phonon (the low-energy peak, open markers) at the O *K* edge. The dispersion is extracted from the fitting. Phonon intensity is the area of the curve fitting each phonon mode.

In Fig. 1*C*, we highlight an excitation at ∼60 meV whose intensity is enhanced for *q*_//_ between Q_CDW_ and the zone boundary. At the O *K* (Fig. 1*D*) the excitation is much weaker in comparison to the CDW peak. To quantify the *q*_//_ dependence of the inelastic component, we fitted the quasielastic peak and then subtracted it from the RIXS spectra (*Materials and Methods* and *SI Appendix*, Figs. S5 and S6). Fitting examples of the excitation spectra at the Cu *L*_3_- and O *K* edges are shown in Fig. 2 *A* and *B*, respectively. The *q*_//_-dependent inelastic excitations are displayed in Fig. 2 *C* and *D* with selected spectra depicted in Fig. 2 *E* and *F* covering *q*_//_ of 0.2 to 0.3 r.l.u. Most notably, the line profile of the inelastic spectra at the Cu *L*_3_ edge is single-peaked while a double-peaked structure appears at the O *K* edge. To elucidate the latter, we collected higher-energy resolution RIXS spectra from the same sample and were able to clearly resolve the lower-energy peak (*SI Appendix*, Fig. S7). The dispersion and integrated spectral weight were extracted and summarized in Fig. 2 *G*–*J*. At the Cu *L*_3_ resonance (Fig. 2*G*), the inelastic peak is located around 65 meV at small *q*_//_ then softens in the *q*_//_ range of 0.2 ∼ 0.4 r.l.u., with a broad “dip” developing at about 50 meV near Q_CDW_. The extracted dispersion matches well with those obtained using π-polarized light and they are reminiscent of the phonon softening observed in Bi2212 (17) (*SI Appendix*, Fig. S8). Although the softening wavevector is close to Q_CDW_ in Bi2201 and Bi2212, a recent RIXS study of CDW correlations in LSCO shows that the phonon softening develops at Q > Q_CDW_, possibly implying a delicate relationship between the momentum of the phonon softening and the CDW wavevectors (19). In Fig. 2*H*, the integrated intensity has a nonmonotonic increase as a function of q_//_ with a maximum around 0.35 r.l.u. Noticeably, both the dispersion and the intensity profiles of the inelastic peak agree very well with that of the Cu–O bond-stretching phonon branch [the half-breathing Δ_1_ mode along the (100) direction] in the underdoped Bi2212 (17). The intensity enhancement at wavevectors larger than Q_CDW_ resembles the Fano interference effect of the bond-stretching phonon in the underdoped Bi2212 (17). These observations suggest the existence of the dispersive CDW excitations that interact with the bond-stretching phonon in UD23. We highlight that the phonon intensities measured by RIXS and IXS are very different: the former is proportional to the strength of the EPC in the reciprocal space while the latter measures the phonon self-energy (15–17, 20, 21).

Concerning the excitation at the O *K* edge, the high-energy peak exhibits a downward dispersion (Fig. 2*I*) and a rising intensity (Fig. 2*J*) with increasing q_//_, akin to the bond-stretching phonon branch observed at the Cu *L*_3_ edge. The low-energy peak, however, is centered at ∼30 meV showing little dispersion (Fig. 2*I*). Its intensity (Fig. 2*J*) gradually decreases from *q*_//_ of 0.1 to 0.2 r.l.u., before abruptly forming a dome-shaped enhancement peaked around Q_CDW_ of 0.25 r.l.u. The central energy of the low-energy branch is comparable to that of a bond-buckling phonon in YBa_2_Cu_3_O_7_ (YBCO) identified by INS (22). In fact, a RIXS study on the undoped compound NdBa_2_Cu_3_O_7_ (NBCO) revealed the in-phase A_1g_ mode of the bond-buckling phonon at ∼30 meV in accord with our data (23). RIXS experiments on NBCO and the model calculations both confirm that the EPC of the in-phase bond-buckling phonon decreases from the Brillouin zone center toward the zone boundary while the EPC of the bond-stretching phonon shows the opposite trend (20, 23, 24). Comparing RIXS results between UD23 and NBCO, we found that the q-dependent EPC of the high-energy bond-stretching phonon agrees reasonably well. But, an apparent discrepancy exists for the low-energy bond-buckling phonon: the dome-shaped intensity enhancement around Q_CDW_ in UD23 is in stark contrast to the monotonically decreasing EPC in parent NBCO (23). The anomalous softening of the buckling phonon in YBCO was suggested to be associated with a charge-density modulation, which is now understood to be omnipresent in underdoped cuprates (22). The giant intensity anomaly of the buckling mode in UD23 may, therefore, reflect an interplay with the dispersive CDW excitations.

**Fig. 3.**
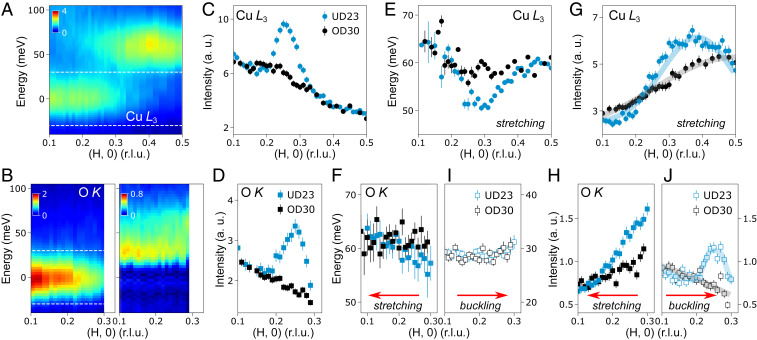
CDW and phonons studies in overdoped OD30. (*A* and *B*) Cu *L*_3_- and O *K* RIXS intensity map as a function of energy and *q*_//_ along the (H, 0) direction, respectively. The map on the right-hand side of *B* is the residual excitations after subtraction of the fitted elastic peaks. (*C* and *D*) Integrated intensities of the quasielastic peaks at the Cu *L*_3_- (black dots) and O *K* (black squares) within the energy window (±30 meV) defined by the two white dashed lines in *A* and *B*. Integrated elastic peaks intensities in UD23 from the same energy range are shown in blue for comparison. (*E*) Dispersion of the bond-stretching phonon excitations at the Cu *L*_3_, in OD30 (black dots) extracted from the fitting. (*F* and *I*) Dispersion of the bond-stretching phonon (black filled squares) and bond-buckling phonon (black open squares) excitations at the O *K* edge. For *E*, *F*, and *I*, phonon peak positions extracted from UD23 sample are displayed in blue for comparison. (*G*) Integrated intensity of the bond-stretching phonon at the Cu *L*_3_ edge within the same energy window (0–100 meV) as for UD23 sample after the removal of the elastic peak. (*H* and *J*) Bond-stretching and bond-buckling phonon intensities through the integration of the area of each fitted phonon mode at O *K* edge. For *G*, *H,* and *J*, phonon intensities in UD23 are displayed in blue for comparison. Fits of the phonon intensities are superimposed on top of experimental data in *G* and *J*.

To elucidate the anomalous intensity enhancement of the buckling phonon, we surveyed the other parts of the phase diagram by investigating a slightly overdoped Bi_2_Sr_1.8_La_0.2_CuO_6+δ_ superconducting compound (T_c_ = 30 K, OD30), anticipating that the CDW correlations and EPC may be rather different from its underdoped counterpart. In Fig. 3 *A* and *B*, Cu *L*_3_- and O *K* RIXS spectra of OD30 are plotted as a function of q_//_ = (H, 0). No scattering peak is observed in the quasielastic region at either edge across the whole accessible q_//_ range. To expand on this observation, we studied RIXS under various configurations including along the (−H, 0) direction, the (H, H) direction, and in off-resonance conditions (*SI Appendix*, Fig. S9). None of them reveal any CDW signatures, making this system distinct from underdoped and the extremely overdoped Bi2201, where the CDW was found (6, 25). We illustrate these remarkable results in Fig. 3 *C* and *D*. In contrast to the well-defined CDW peak in the UD23 sample, the integrated intensity of the quasielastic peak in OD30 has a simple backgroundlike profile demonstrating the complete obliteration of CDW correlations.

Correspondingly, the phonon excitations manifest differently in the absence of CDW correlations. First, the bond-stretching phonon softening is largely suppressed, regardless of whether it is probed on the Cu- (Fig. 3*E*) or O sublattices (Fig. 3*F*). Second, the EPC anomaly of the bond-stretching mode, i.e., the broad intensity enhancement at q_//_ > Q_CDW_, diminishes in both sublattices, resulting in a simple upward increase (Fig. 3 *G* and *H*). Similarly, the intensity of the bond-buckling phonon is significantly altered with no appreciable change in its dispersion (Fig. 3*I*). The entire dome-shaped enhancement vanishes in OD30 and a monotonically decreasing intensity profile is formed as a function of q_//_ (Fig. 3*J*), opposite to the trend of the bond-stretching phonon.

**Fig. 4.**
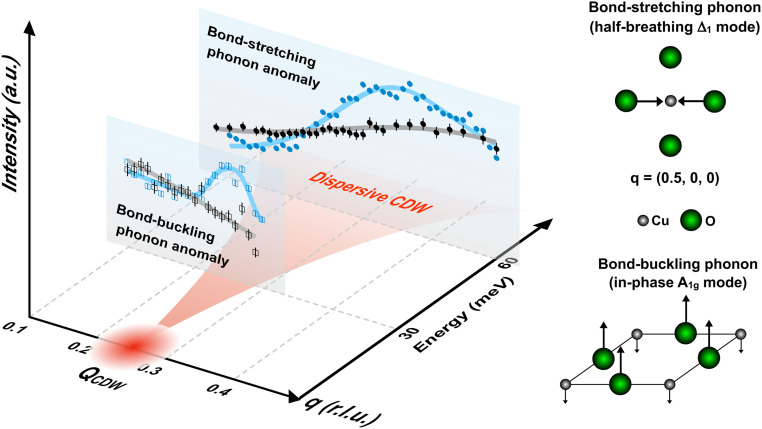
Quasistatic CDWs, their dispersive excitations, and concomitant electron-phonon anomalies. A sketch of the quasistatic CDW, the dispersive CDW excitations, and their interplay with the bond-stretching and the bond-buckling phonon modes. Blue and gray markers are the experimental phonon intensities of UD23 and OD30 samples, respectively, as shown in Fig. 3 *G* and *J*. Solid lines are fitting results. Sketches on the right illustrate the bond-stretching phonon (the half-breathing Δ_1_ mode) at q = (0.5, 0, 0) and the bond-buckling phonon (in-phase) A_1g_ mode.

It becomes clear now that the momentum-dependent EPC of each phonon branch in the absence of CDW correlations in OD30 coincides with that of the parent NBCO where the bond-stretching and the bond-buckling phonon intensities scale with sin^2^(πH) and cos^2^(πH) functions, respectively (20, 23, 24). We found that these functional forms describe the observed momentum-dependent phonon intensities quite well (Fig. 3 *G* and *J*). The good consistency between OD30 and NBCO provides compelling evidence that the phonon intensity anomaly, in UD23, is not owing to straightforward increase in the EPC, but rather a complex reflection of the Fano interference effect induced by the dispersive CDW excitations. In Fig. 4, we illustrate a comprehensive picture of the quasistatic CDWs, the dispersive CDW excitations, and concomitant electron-phonon anomalies in the momentum-energy space. Emanating from the quasistatic CDWs, the CDW excitations quickly disperse and intersect firstly with the bond-buckling phonon at low energy. The narrow CDW excitations in the momentum space result in a dome-shaped phonon intensity enhancement closely confined around Q_CDW_. After reaching higher energy and greater q_//_ (>Q_CDW_), the dispersive CDW excitations significantly broaden in the momentum space and intersect with the bond-stretching phonon inducing a diffused intensity anomaly. The intensity enhancement at q_//_ > Q_CDW_ side is due to the momentum-dependent EPC of the bond-stretching phonon. It is worth mentioning that excitations of a conventional CDW can be gapped by the periodically distorted crystal lattice or by impurities (26). Similarly, the dispersive CDW excitations in Bi2201 may exhibit a gap falling below the current detection limit.

The strong intensity anomalies of the buckling and stretching phonons in UD23 allow us to deduce the characteristic velocity of the dispersive CDW excitations. To do so, we first fitted the momentum-dependent phonon intensities and retrieved the wavevector (Q_A_) of the maximal intensity anomaly (Fig. 3 *G* and *J* and *SI Appendix*, Fig. S10). By connecting Q_A_ = 0.34 r.l.u. with Q_CDW_ = 0.259 r.l.u. at the Cu *L*_3_ edge, we extracted the velocity of the CDW excitations, V_CDW-stretching_ ∼0.45 ± 0.05 eV Å, close to the bond-stretching phonon (∼60 meV). Near the bond-buckling phonon (∼30 meV), we joined the intensity anomalies at Q_A_ = 0.238 and 0.267 r.l.u. with Q_CDW_ = 0.25 r.l.u. from the O *K* edge, and obtained an averaged velocity of the CDW excitations, V_CDW-buckling_ ∼1.3 ± 0.3 eV Å. Remarkably, V_CDW-buckling_ is about four times larger than V_CDW-stretching_, demonstrating unambiguously that the CDW excitation disperses steeply after arising from the quasistatic CDW, then gradually flattens at higher energy. Overall, the trend extracted by the bond-stretching and the bond-buckling phonon describes funnel-shaped dispersive CDW excitations as highlighted in Fig. 4. We notice that the V_CDW-stretching_ in Bi2201 is comparable to that of the underdoped Bi2212 (∼0.6 ± 0.2 eV Å) at the energy ∼60 meV (17). Interestingly, at the energy ∼30 meV, the deduced CDW excitations velocity in Bi2201 is close to that of the electron band dispersion, ∼1.7 ± 0.2 eV Å, retrieved from angular-resolved photoemission data in underdoped Bi2212 (27). The similarity of the velocities may indicate comparable self-energies between the ordinary and the periodically modulated charge carriers. However, this connection should not be simply viewed as an evidence of the weak-coupling (i.e., Fermi surface nesting) picture to describe the emergence of CDWs correlations. Rather, it is crucial for theoretical models to take into account the velocity values for the description of the CDWs in cuprates.

Lin et al. recently reported little signature of dispersive CDW excitations in LSCO compounds despite the phonon-softening across a wide doping range (19). The bond-stretching phonon intensities, with and without the presence of CDW correlation, show similar momentum dependence, in contrast to our Bi2201 RIXS data (Fig. 3 *G*, *H*, and *J*). As the bond-buckling phonon intensity anomaly is much more confined around Q_CDW_ differentiating drastically from the bare EPC, we corroborate the highly sensitive O *K* RIXS in the detection of the coupling of CDW excitations with phonons and its complementary with Cu *L* RIXS.

The coexistence of CDW at the Cu and O sublattices in Bi2201 demonstrates that the modulated charge density carries both Cu-3*d* and O-2*p* orbital character. REXS studies on LBCO and YBCO showed that the CDW order has *s′-* or *d*- wave symmetry which depict a bond-centered charge order naturally explaining the projection onto Cu and O sublattices (14, 28). Given the correlation length of the CDW in Bi2201 is more than an order of magnitude shorter than in LBCO (29), we suggest that the multiorbital nature is universal to the CDWs in all hole-doped cuprates. From a theoretical perspective it is unclear whether the CDWs can be captured properly using a single-band model given the significant oxygen character of the density modulation and the phonons involved. Our work, therefore, highlights the need to use multiorbital Hubbard models (specifically three-bands Hubbard models) to describe the cuprates in terms of electron dynamics and orders (13, 30–32).

Our findings on the rich interplay between the multiorbital CDW and the electron-phonon anomalies in Bi2201 substantiate the existence of funnel-shaped CDW excitations dispersing in the energy-momentum space. This is a major step forward in characterizing the dispersive CDW excitations comparing to the previous study on Bi2212, which was solely based on the interference effect from a single-phonon branch (17). The dispersive CDW excitations are in line with the short-range dynamical charge-density fluctuations found in a large portion of the phase diagram in YBCO, postulating its ubiquity in cuprates (12). Further experiments aimed at uncovering dynamical CDW excitations in different cuprates may help elucidate the relevance of CDWs for the anomalous normal state and the unconventional superconducting properties. Concerning the underlying mechanism of CDWs, the most recent CDW studies on YBCO and LSCO add mounting evidence that the Fermi-surface nesting alone is unlikely to be the primary driving force (12, 19). Moreover, the fact that the CDW dynamics interfere with the phonons points toward an important role of the EPC in the formation of the CDWs (33–36). As suggested by refs. 20, 21, 23, 37, 38, the phonon intensity measured by RIXS is scaled to *M*^2^, where *M* is the EPC matrix element. A simple comparison of phonon intensities between UD23 and OD30 (Fig. 3 *G*, *H*, and *J*) would imply that *M* at momenta far away from the phonon anomalies is comparable between two Bi2201 compounds. However, determining *M* at Q_CDW_ faces difficulties as the RIXS intensity is not a simple proportion to *M*^2^ but rather reflects a complex interplay with CDW excitations. It is currently challenging to extract reliably the EPC at CDW wavevector and a much more sophisticated theoretical modeling is required in the future.

## Conclusion

We combined high-resolution RIXS at the O *K*- and Cu *L*_3_ edges to study the quasistatic CDWs, the collective CDW excitations, and the electron-phonon coupling in Bi2201. The quasistatic CDWs are present at both Cu and O sublattices and carry comparable periodicity and correlation length implying their multiorbital nature to be universal in hole-doped cuprates. Both the bond-stretching and the bond-buckling phonons exhibit strong anomalies concomitant to CDWs. The confined intensity anomaly of the bond-buckling phonon, together with the diffused intensity enhancement of the bond-stretching phonon, uncovered unambiguously funnel-shaped dispersive CDW excitations. The significant interference effects suggest that CDWs are intimately connected to the electron-phonon coupling, which needs to be considered as a crucial element for the underlying mechanism leading to CDWs in cuprates.

## Materials and Methods

### Sample Growth and Characterization.

High-quality single crystals of UD23 and OD30 Bi_2_Sr_2−*x*_La_*x*_CuO_6+δ_ cuprates with *x* = 0.6 and 0.2, respectively, were grown by the traveling-solvent floating-zone method. The as-grown samples were annealed at 650 °C in oxygen atmosphere for 2 d to improve sample homogeneity. The samples were precharacterized and aligned using Laue diffraction prior to RIXS experiments (*SI Appendix*, Fig. S1). Superconducting transition temperature T_c_ are 23 and 30 K for UD23 and OD30 (*SI Appendix*, Fig. S1) which are consistent with a doping concentration of *p* ∼ 0.13 and ∼0.18, respectively (39).

### High Resolution RIXS Measurements on UD23 and OD30.

High-resolution RIXS experiments were performed at the I21-RIXS beamline at Diamond Light Source, United Kingdom. Samples were cleaved in air prior to the transfer into the sample load-lock vacuum chamber. The experimental geometry is sketched in *SI Appendix*, Fig. S2. All samples were aligned with the surface normal (001) lying in the scattering plane. X-ray absorption was measured using the total electron yield method by recording the drain current from the samples. For RIXS measurements, linear σ- and π-polarized X-rays were used. The total energy and momentum resolution were about 40 meV (26 meV) (FWHM) and ±0.012 Å^−1^ (±0.006 Å^−1^) at the Cu *L*_3_- (O *K)* edge, respectively. To enhance the RIXS throughput, a special paraboloidal mirror is implemented in the main vacuum chamber. The RIXS spectrometer was positioned at a fixed scattering angle of 154° resulting in a maximal total momentum transfer value Q, of ∼0.92 Å^−1^ (0.52 Å^−1^) at the Cu *L*_3_- (O *K)* edge. The projection of the momentum transfer, q_//_, in the *a*-*b* plane was obtained through varying the grazing incident angle of the sample owing to the quasi-2D CDW in Bi2201 system. We use the pseudotetragonal unit cell with *a* = *b* = 3.86 Å and *c* = 24.69 Å for the reciprocal space mapping. The momentum transfer **Q** is defined in r.l.u. as **Q** = Ha* + Kb* + Lc* where a* = 2π/a, b* = 2π/b, and c* = 2π/c. All measurements were done at 20 K under a vacuum pressure of about 5 × 10^−10^ mbar. To confirm the observation of CDW, the RIXS measurements were repeated on a second sample for UD23 with the same experimental setup. Detailed results are summarized in *SI Appendix*, Fig. S4.

### Data Analysis and Data Fitting.

All RIXS spectra have been normalized by the counting time and corrected for self-absorption effects through the procedure described in *SI Appendix*. The zero-energy positions of RIXS spectra were determined by comparing to reference spectra recorded from the amorphous carbon tapes next to the sample for each q_//_ position. They were finely adjusted through the Gaussian fitting of each elastic peak. The intensity of CDW as a function of q_//_ results from the integration of spectra within an energy window of ± 30 meV and was fitted with a Gaussian profile and a power-law function as a background. To quantify the phonon excitations, the fitting model for the Cu *L*_3_ spectra (from −100 up to 800 meV) consists of a Gaussian (elastic peak) with a width constrained to the instrumental energy resolution, a Lorentzian (bond-stretching phonon), a damped harmonic oscillator model to account for the paramagnon at high energy and a linear background. For the O *K* RIXS data where the elastic peaks are generally much stronger, an instrumental energy resolution-limited Gaussian function is firstly used to fit the spectra ranging from −100 to 25 meV, then two Gaussians (for the buckling and bond-stretching phonon modes) and a linear trend (for the background) are applied to fit the residual spectra from −100 to 150 meV after removing the fitted elastic peaks. We note that the fitted phonon parameters in the O *K* spectra are generally consistent with a global fitting containing three Gaussian functions, except the central position of the bond-buckling mode is ∼10 meV lower than the fitted central position of ∼30 meV presented in the main text. Error bars of the phonon dispersions are determined by a combination of the uncertainty of determining the zero-energy position and SDs from the fit. For the integrated RIXS intensities of CDW and phonons, error bars are based on the noise level of measured spectra.

### Data Availability.

All relevant data have been deposited in the Zenodo repository at http://doi.org/10.5281/zenodo.3890415; all materials are available in the *SI Appendix*.

## Supplementary Material

Supplementary File
