# Supplementary material for: Multiorbital charge-density wave excitations and concomitant phonon anomalies in Bi2Sr2LaCuO6+δ
Source: Proc Natl Acad Sci U S A. 2020 Jun 25;117(28):16219–25. doi: 10.1073/pnas.2001755117 (PMC7368327; doi:10.1073/pnas.2001755117)
Supplement: Supplementary File [file pnas.2001755117.sapp.pdf]

Supplementary Information for

Multiorbital charge-density wave excitations and concomitant phonon anomalies in $Bi_2Sr_2LaCuO_{6+\delta}$

Jiemin Li, Abhishek Nag, Jonathan Pelliciari, Hannah Robarts, Andrew Walters, Mirian Garcia-Fernandez, Hiroshi Eisaki, Dongjoon Song, Hong Ding, Steven Johnston, Riccardo Comin, Ke-Jin Zhou

Ke-Jin Zhou
Email: kejin.zhou@diamond.ac.uk

This PDF file includes:

- Supplementary text
- Figures S1 to S10
- Tables S1
- SI References

www.pnas.org/cgi/doi/10.1073/pnas.2001755117

Supplementary Information Text

Materials

we focused on Bi2201 superconductors with two different doping levels: (1) $Bi_2Sr_{1.4}La_{0.6}CuO_{6+\delta}$ (UD23), p ~ 0.13, Tc ~ 23 K; (2) $Bi_2Sr_{1.8}La_{0.2}CuO_{6+\delta}$ (OD30), p ~ 0.18, Tc ~ 30 K, as indicated by the arrows shown in Fig. S1D. The measured Laue patterns of the samples are displayed in Fig.S1A and Fig. S1B. The superconducting transition temperature, Tc, were determined from the magnetization measurements shown in Fig. S1C and S1D. Please note that 10 Oe is already a low field limit therefore the use of the magnetic field values (10 and 1 Oe) does not make considerable difference for determining Tc between two samples. A set of super-structural (SS) points due to the structural distortion of BiO planes are clearly resolved and used as the reference to orient samples.

RIXS scattering geometry

Fig. S2A shows a sketch of the RIXS experimental scattering geometry. Samples were mounted such that the *a*-axis and *c*-axis lay in the horizontal scattering plane while the *b*-axis was perpendicular to the scattering plane. During the measurements, the spectrometer was fixed to Ω = 154° and the projection of the momentum transfer was obtained through varying θ angle around the sample b-axis. Both σ and π polarized incoming X-rays were employed to probe samples with no polarization analysis for the outgoing scattered X-rays. To get a clean sample surface for X-ray measurements, we cleaved the samples in air prior transferring into the load-lock vacuum chamber. Throughout the whole experiments, the reciprocal space is defined with a unit cell of *a* = *b* = 3.86 Å and *c* = 24.69 Å. We display all the data as reciprocal lattice units. Accessible momentum space in the first Brillouin Zone at the Cu L_3-edge (green shaded circle) and the O *K*-edge (magenta shaded circle) are shown in Fig. S2B. The measurements were done mainly along the (H, 0), (0, H) and (H, H) directions.

Self-absorption correction for RIXS spectra

Self-absorption can distort the spectral line profile severely. To eliminate this effect, we follow the procedure described in Ref. S1. Considering that the elastic peak and the low-energy phonon do not involve spin-flip processes in which the X-ray polarization is preserved, *i.e.*, $I_{\sigma\to\pi} = I_{\pi\to\sigma} = 0$, the intensity correction can be described as:

$$I_{\sigma}{}^{corr} = I_{\sigma\to\sigma}{}^{corr} = I_{\sigma}/C_{\sigma\to\sigma} = I_{\sigma} \times \left(\mu_{i\sigma} + \mu_{f\sigma} \times \frac{-\vec{k}_i \cdot \vec{n}}{\vec{k}_f \cdot \vec{n}}\right) = I_{\sigma} \times f_a \times \left(1 + \frac{sin\theta}{sin(\Omega-\theta)}\right),$$

$$I_{\pi}{}^{corr} = I_{\pi\to\pi}{}^{corr} = I_{\pi}/C_{\pi\to\pi} = I_{\pi} \times \left(\mu_{i\pi} + \mu_{f\pi} \times \frac{-\vec{k}_i \cdot \vec{n}}{\vec{k}_f \cdot \vec{n}}\right)$$

$$= I_{\pi} \times \left((f_a sin^2\theta + f_c cos^2\theta) + (f_a sin^2(\Omega-\theta) + f_c cos^2(\Omega-\theta)) \times \frac{sin\theta}{sin(\Omega-\theta)}\right)$$

Here, μ_i (μ_f) and $\vec{k}_i$ ($\vec{k}_f$) characterize the absorption coefficient and the direction of the incoming (outgoing) X-rays, respectively. $\vec{n}$ is the surface normal direction, f_a and f_c are the Cu or O atom scattering factor along *a* or *c*-axes which can be extracted from the XAS with $f_a \gg f_c$ due to the quasi-two-dimensional nature of the sample. It is clear that the self-absorption correction for σ polarized RIXS spectra is only related to the scattering geometry and scaled by the in-plane scattering factor f_a. Whereas for the π polarized spectra, both f_a and f_c contribute to the correction. The self-absorption correction was applied to all RIXS data presented in the paper.

CDW excitations in various configurations

We present the observation of the CDW excitation in various experimental configurations alongside the results shown in the main text. In Fig. S3, we show the RIXS map excited using the π polarized incident X-rays along the (H, 0) direction, RIXS map excited using the σ polarized incident X-rays along the (0, H) direction, and RIXS map excited using the σ polarized incident X-rays along the (H, H) direction. We see clear CDW scattering peak along the (H, 0) and (0, H) directions regardless

the polarization of the incident X-rays. However, no CDW scattering peak is formed along the (H, H) direction consistent to the RXS study on Bi2201 compounds (S2). Integrated intensity within the quasi-elastic region (± 30 meV) defined by the white dashed lines was summarized in Fig. S3D. CDW peak along the (H, 0) or the (0, H) direction has a characteristic wavevector at $q_{//}$ ~ 0.26 *r.l.u.*.

Repeatability of the presence of the CDW peak in UD23

To confirm the observation of CDW at both the Cu L_3- and O *K*- edges in UD23, we repeated the measurements on a second sample of UD23 which show again CDW scattering peak at both edges. Using the fitting analysis described in the Materials and Methods of the main text, we show the fitting results in Table S1 and the integrated intensity of the quasi-elastic peak (± 30 meV) in Fig. S4. The error bars shown in the table are the convolution of the instrumental momentum resolution and the standard deviation of the fitting errors.

Fittings of the bond-stretching and the bond-buckling phonons spectra

We show detailed fitting results of the bond-stretching and the bond-buckling phonons in $q_{//}$ dependent spectra in UD23 at the Cu L_3 and the O *K* in Figs. S5, and S6, respectively. For the Cu L_3-edge data, a Gaussian function with the instrumental resolution is used for fitting the elastic peak, a Lorentzian function is applied to the bond-stretching phonon, the tail of paramagnon is fitted by a damped harmonic oscillator multiplied by the Bose factor, and a linear function is used for fitting the general background. For the O *K*-edge data, two Gaussian functions are used to fit the residual spectra after subtracting the fitted elastic peak to track the bond-stretching and the bond-buckling phonon modes.

Ultra-high energy resolution O *K*-edge RIXS of UD23

The fitting analysis for the O *K* RIXS spectra of UD23 shows that the phonon excitations contain a two-peak structure although the lower-energy phonon is less visible comparing to the higher-energy phonon peak in the raw data. To confirm the observation, we re-measured the same UD23 sample using a higher energy resolution (FWHM = 18 meV) set-up than the normal energy resolution (FWHM = 26 meV) set-up used for the main measurements. Figure S7 shows the comparison of the raw RIXS spectra at $q_{//}$ of (0.23, 0). A low energy phonon excitation below 50 meV is clearly resolved in the higher energy resolution RIXS spectrum.

Extracted dispersion of the bond-stretching phonon in UD23 at Cu L_3

Using the same fitting analysis described in the Materials and Methods of the main text, we fitted the excitations at the Cu L_3-edge of UD23 measured along the (H, 0) direction using π polarized X-rays and along the (H, H) using σ polarized X-rays. Extracted dispersion of the bond-stretching phonon along the (H, 0) shows quite good consistency between σ and π polarizations. In particular, both develop a softening near Q_{CDW} despite totally different $q_{//}$-dependent phonon intensity distributions (Fig. S3A vs Fig. S3B). The extracted phonon dispersion along the (H, H) direction shows no softening with slightly a higher excitation energy comparing to that along the (H, 0) direction. Results are shown in Fig. S8.

Detailed examination of the quasi-elastic peak in OD30 at Cu L_3- and O *K*- edges

At the resonance of the Cu L_3-edge (931.6 eV) and the hole peak (528.4 eV) of O *K*-edge in OD30 sample, no CDW is observed as shown in Fig. 3. To further explore the existence of the CDW, we performed the Cu L_3 RIXS measurements at the resonance (931.6 eV) along the (-H, 0) (Fig. S9A), at an energy off-resonance (932 eV) along the (H, 0) direction (Fig. S9B), and at the resonance (931.6 eV) along the (H, H) direction (Fig. S9C). For O *K* RIXS, we collected data at an energy off-resonance of the hole peak (528.1 eV) (Fig. S9E). Figures S9D and Fig. S9F summarize the integrated intensity of the quasi-elastic peak within the energy window (± 30 meV) defined by white dashed lines at the Cu L_3 and O *K* edges, respectively. None of them shows any signature of CDW scattering peak. CDW peak from UD23 is shown in both Fig. S9D and Fig. S9F for comparison.

Fittings of the momentum-dependent intensities of the bond-stretching and the bond-buckling phonons in UD23

To extract the velocity of dispersive CDW excitations in UD23, we fitted the momentum-dependent intensities of the bond-stretching (bond-buckling) phonon mode obtained from the Cu L_3 (O *K*) RIXS. We know that the momentum-dependent intensities of the bond -stretching and -buckling phonons in OD30 follow $\sin^2(\pi H)$ and $\cos^2(\pi H)$ functions, respectively. For the bond-stretching phonon in UD23, we use $A\sin^2(\pi H) + C$, where A and C are constants, to describe the background signal and a Gaussian to account for the anomalous phonon profile. Likewise, for the bond-buckling phonon, $A'\cos^2(\pi H) + C'$ function is used to fit the background and two Gaussians are used to fit two phonon anomalies. We obtained the bond-stretching phonon anomaly at $Q_A = 0.34 \pm 0.007$ *r.l.u.*. For the bond-buckling phonon, the two anomalies are at $Q_A = 0.238 \pm 0.004$ *r.l.u.*, and $Q_A = 0.267 \pm 0.004$ *r.l.u.*. The velocity of dispersive CDW excitations near the bond-buckling phonon is an averaged value based on two phonon anomalies. Fittings are shown in Fig. S10.

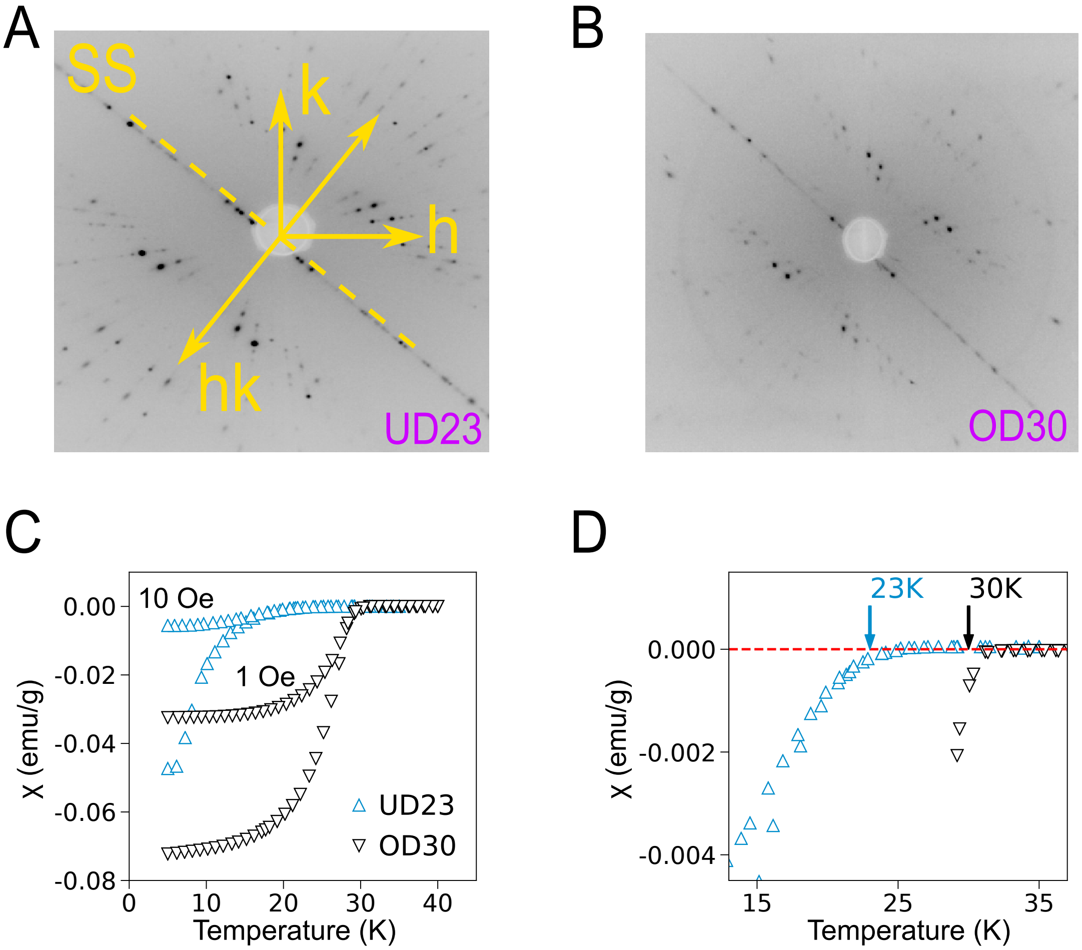

Figure S1. Sample Information. **A** and **B**, The Laue patterns of UD23 and OD30 samples, respectively. The *h* and *hk* denotes (H, 0) and (H, H) direction, respectively. The super-structure (SS) diffraction points of BiO structural distortion is perpendicular to the *hk* direction. **C** and **D**, Magnetization results for UD23 and OD30 samples.

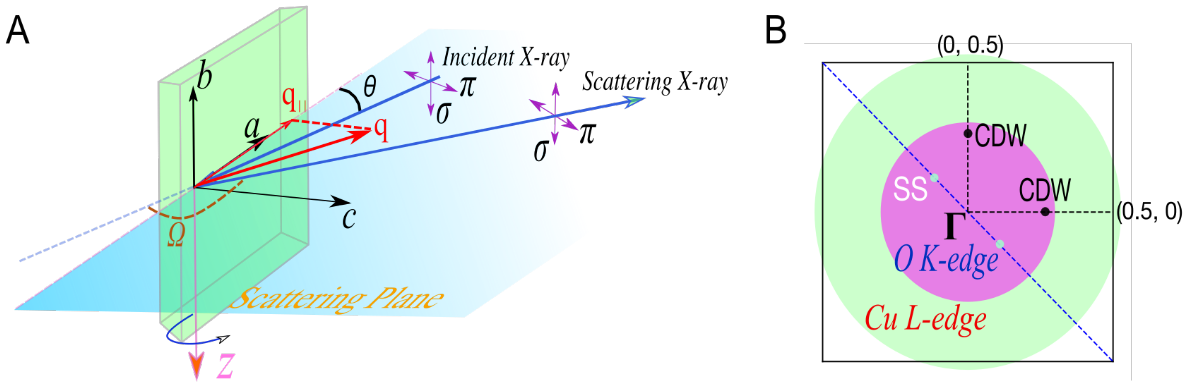

Figure S2. RIXS experimental set-up. A, Scattering geometry. Blue arrows define the incident and scattered X-rays. Purple cross illustrates the linear polarization of the incident X-rays. The red arrow, q, denotes the total photon momentum transfer. Its projection onto the samples a-axis is described by $q_{//}$. Ω defines the two-theta angle between the incident and scattered X-ray beam. a, b, and c define the primary sample lattice axes. The in-plane $q_{//}$ projection along the negative direction, *i.e.*, (-H, 0), and the positive direction, *i.e.*, (H, 0), is defined when $\theta<\Omega/2$, and $\theta>\Omega/2$, respectively. **B**, Accessible momentum space at the Cu L_3-edge (green) and the O *K*-edge (magenta) in the first Brillouin Zone. The SS direction is highlighted by a dashed blue line and the CDW wavevectors are highlighted by black dots.

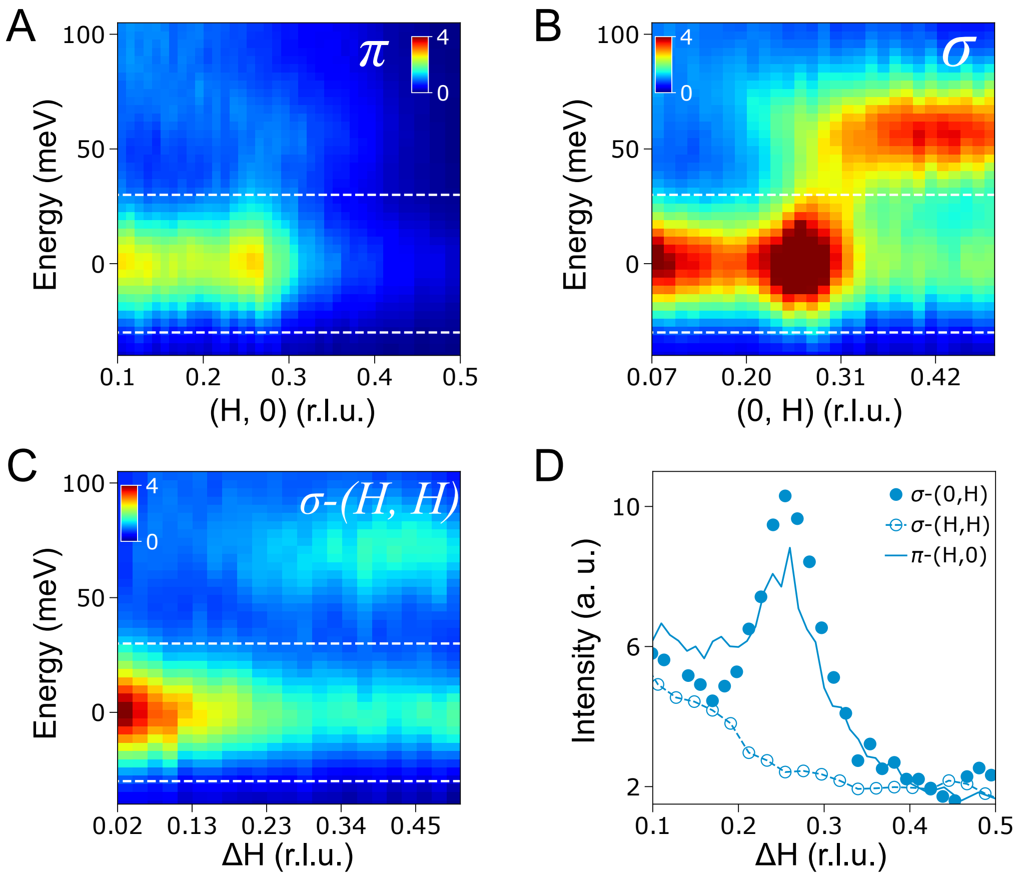

Figure S3. RIXS intensity maps from various configurations. A, Data obtained using π polarized incident X-rays. **B** and **C**, RIXS intensity map using the σ polarized incident X-rays along (0, *H*) and (*H, H*) directions, respectively. **D**, Integrated intensities within the white dashed line defined in **A**, **B** and **C**, as a function of the momentum transfer. Note that the horizontal axes in **C** and **D** are labeled by the absolute value of the in-plane momentum transfer, $\Delta H = \sqrt{H^2 + H^2}$.

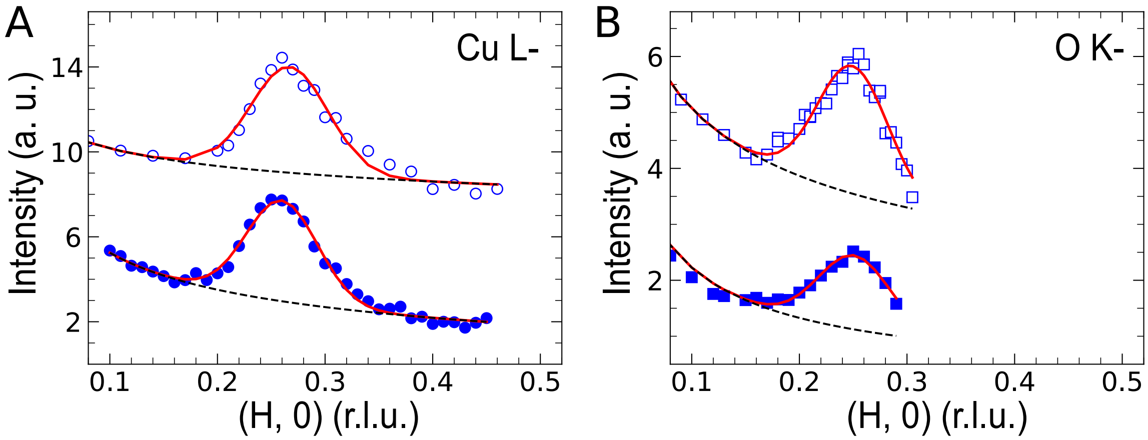

Figure S4. Integrated intensity of the quasi-elastic region of CDW in UD23. The red line is the fitting from a Gaussian and a power-law function (black dashed line). Filled markers are for the Sample 1 shown in the main text, while open markers stand for the Sample 2. Data are shifted vertically for comparison.

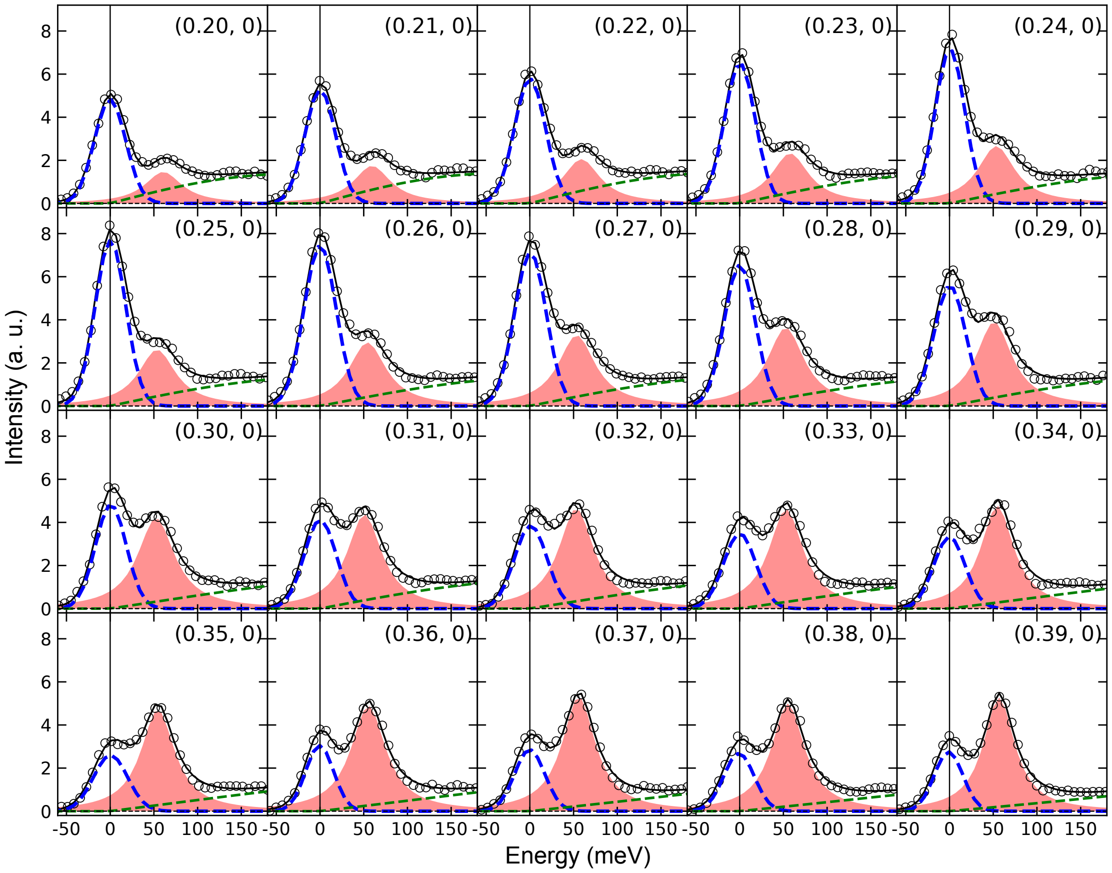

Figure S5. Fittings of the bond-stretching phonon at the Cu L_3-edge in UD23. Blue dashed line is the fit to the elastic peak, the red shaded area represents the fit of the bond-stretching phonon, the green dashed line is the fit to the tail of paramagnon excitations, and the black dashed line stands for the fit of the background.

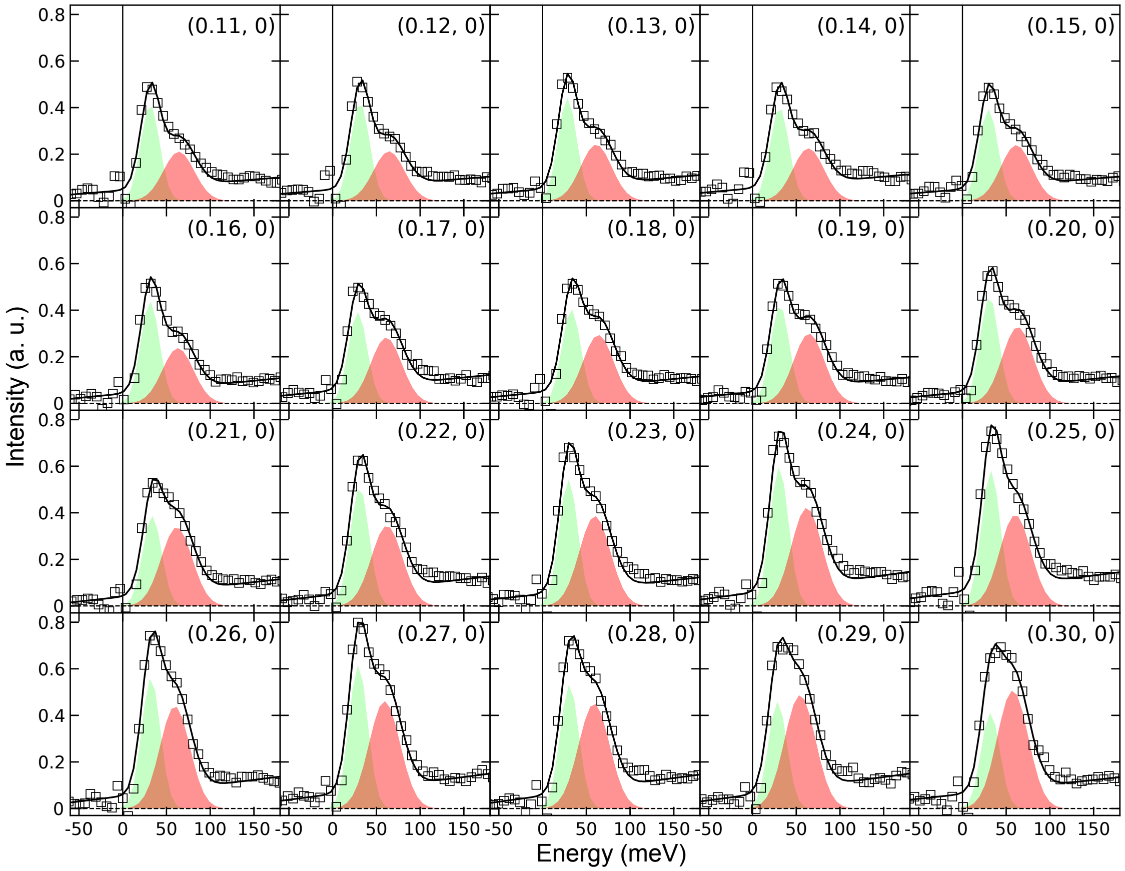

Figure S6. Fitting of the bond-stretching and the bond-buckling phonons at the O *K*-edge in UD23. Green shaded peak represents the fit of the bond-buckling phonon, the red shaded peak represents the fit of the bond-stretching phonon.

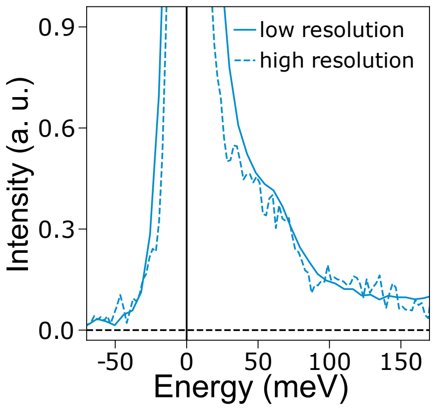

Figure S7. O *K* RIXS data of UD23 sample collected using two energy resolutions. The solid and dashed line represents data collected using an energy resolution (FWHM) of 26 meV and 18 meV, respectively. Both spectra were collected at a fixed momentum transfer $q_{//}$ = (0.23, 0).

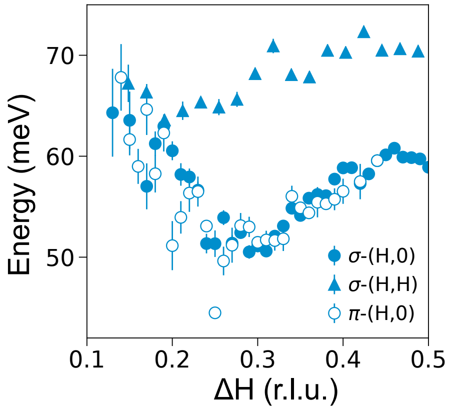

Figure S8. Phonon dispersions of UD23. Extracted phonon dispersion of UD23 sample1 measured along various high symmetry directions in the reciprocal space.

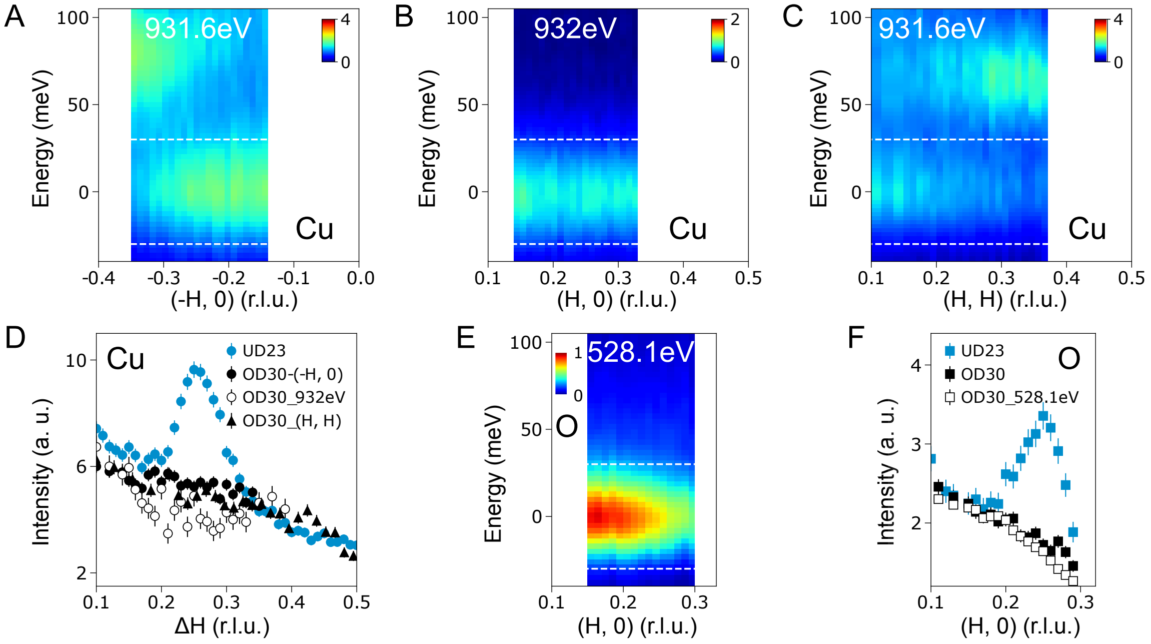

Figure S9. Examination of CDW in OD30 from different experimental configurations. A is along (-H, 0) direction at the resonant photon energy of 931.6 eV. **B** is along the (H, 0) direction at an off-resonant energy of 932 eV. **C** shows the result along the (H, H) direction at the resonant energy of 931.6 eV. **E** shows the off-resonance (528.1 eV) RIXS map at O *K*-edge. **D** and **F,** comparison of integrated quasi-elastic peaks of various configurations at the Cu L_3- and O *K*-edges, respectively. CDW peak of UD23 sample is shown in **D** and **F**.

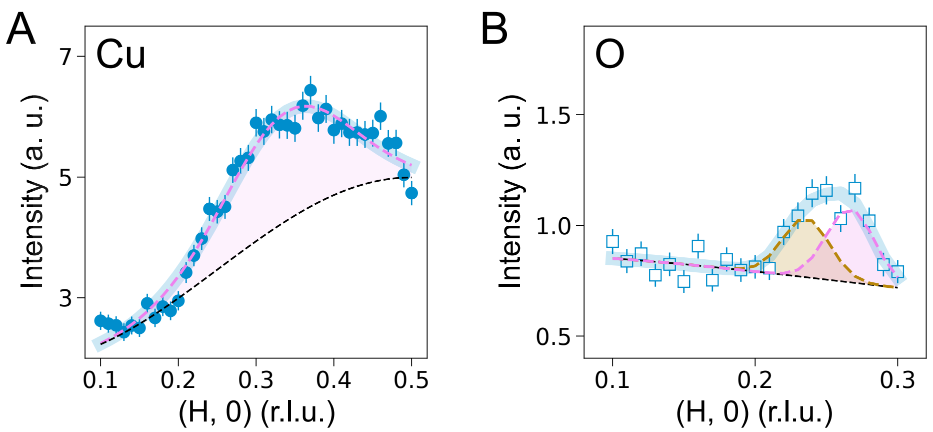

Figure S10. Fitting of momentum-dependent phonon anomalies in UD23. A is the fitting of the bond-stretching phonon mode. The pink dashed line is the fitted Gaussian peak and the black dashed line represents the background. **B** is fitting of the bond-buckling phonon mode. The pink and brown lines are two Gaussian peaks and the black dashed line stands for the background.

Table S1. Repeatability of CDW peak in UD23 samples.

	Cu L_3-edge		O *K*-edge	
	**Q_{CDW} (*r.l.u.*)**	**FWHM (*r.l.u.*)**	**Q_{CDW} (*r.l.u.*)**	**FWHM (*r.l.u.*)**
Sample1	0.259 ± 0.006	0.078 ± 0.006	0.25 ± 0.003	0.076 ± 0.006
Sample2	0.266 ± 0.006	0.084 ± 0.007	0.25 ± 0.003	0.078 ± 0.006

SI References

S1. Kang, M. *et al*. Resolving the nature of electronic excitations in resonant inelastic x-ray scattering. *Phys. Rev. B* **99**, 045105 (2019).

S2. Comin, R. *et al*. Symmetry of charge order in cuprates. *Nat. Mater.* **14**, 796-801 (2015).
